# Supplementary material for: Cellcano: supervised cell type identification for single cell ATAC-seq data
Source: Nat Commun. 2023 Apr 3;14:1864. doi: 10.1038/s41467-023-37439-3 (PMC10070275; doi:10.1038/s41467-023-37439-3)
Supplement: Supplementary file 1 — Supplementary Information [file 41467_2023_37439_MOESM1_ESM.pdf]

Supplementary Tables

Supplementary Table 1. Datasets used in this study

Datasets	Organisms	Tissue	Protocol	No. cells	No. individuals (replicates)	No. cell types
Satpathy et al. [1]	Human	Peripheral blood mononuclear cells (PBMCs)	10X Chromium	21,126	4	6
Granja et al. [2]	Human	PBMCs	10X Chromium	8,302	3 (5)	6
10X PBMC	Human	PBMCs	10X Single Cell Multiome ATAC + Gene Expression	11,909	1	6
FACS PBMC[3]	Human	PBMCs	Flow Cytometry	21,214	1*	5
Lareau et al. [3]	Mouse	Brain	dscATAC-seq	61,558	2	7
Cusanovich et al. [4]	Mouse	Brain	sci-ATAC-seq	18,632	2 (4)	7

Note*: In FACS PBMC dataset, each cell type is extracted from different donors. Here, we consider them as one individual.

Supplementary Figures

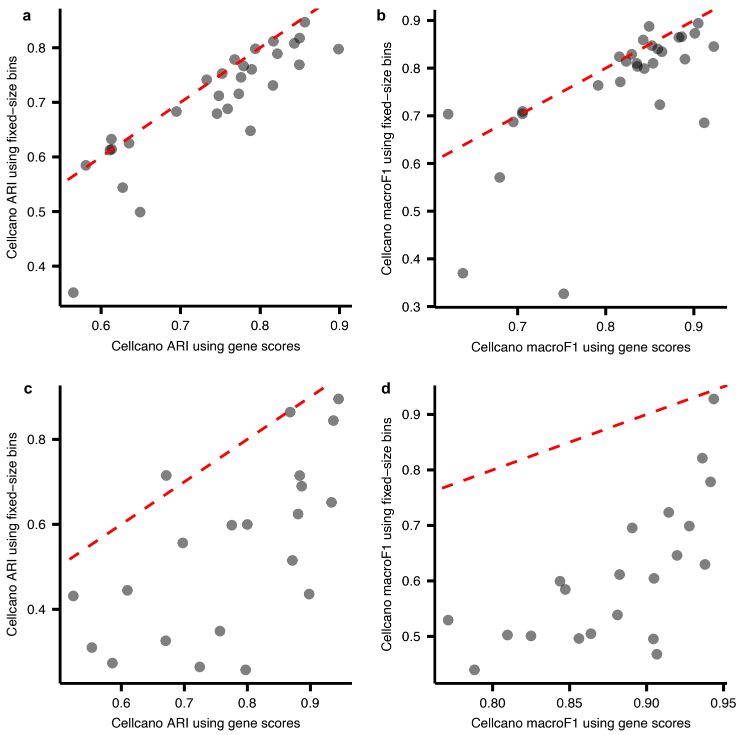

Supplementary Figure 1 (**a**) ARI and (**b**) macroF1 comparisons on $n = 29$ human PBMCs celltyping tasks between Cellcano with genome-wide fixed-size bins as input and Cellcano with gene scores as input. (**c**) ARI and (**d**) macroF1 comparisons on $n = 21$ mouse brain celltyping tasks. The dotted red lines are identity lines.

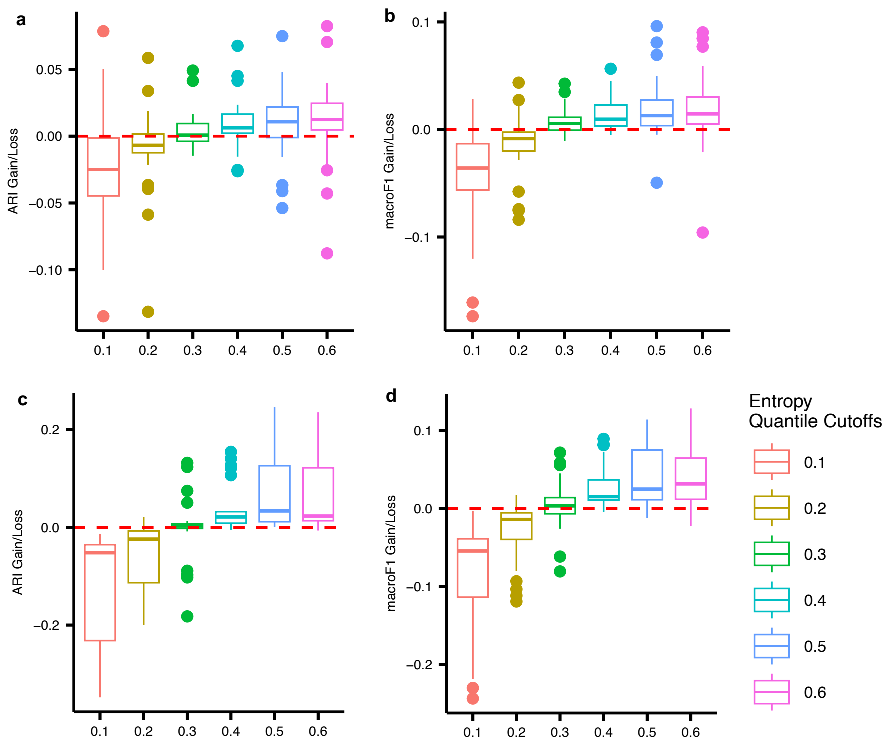

Supplementary Figure 2 Inside the boxes, the middle line indicates the median of the data while the bottom and upper lines indicate the 25th percentile and the 75th percentile of the data. Outside the boxes, the whiskers extend to the minimum and maximum values no greater than 1.5 times interquartile range. Those values outside the range are outliers, which are represented as dots with corresponding colors. (**a**) ARI and (**b**) macroF1 gains/losses using different entropy cutoffs in $n = 29$ human PBMCs celltyping tasks. Each box contains $n = 29$ prediction results. (**c**) ARI and (**d**) macroF1 gains/losses using different entropy cutoffs in $n = 21$ mouse brain celltyping tasks. Each box contains $n = 21$ prediction results.

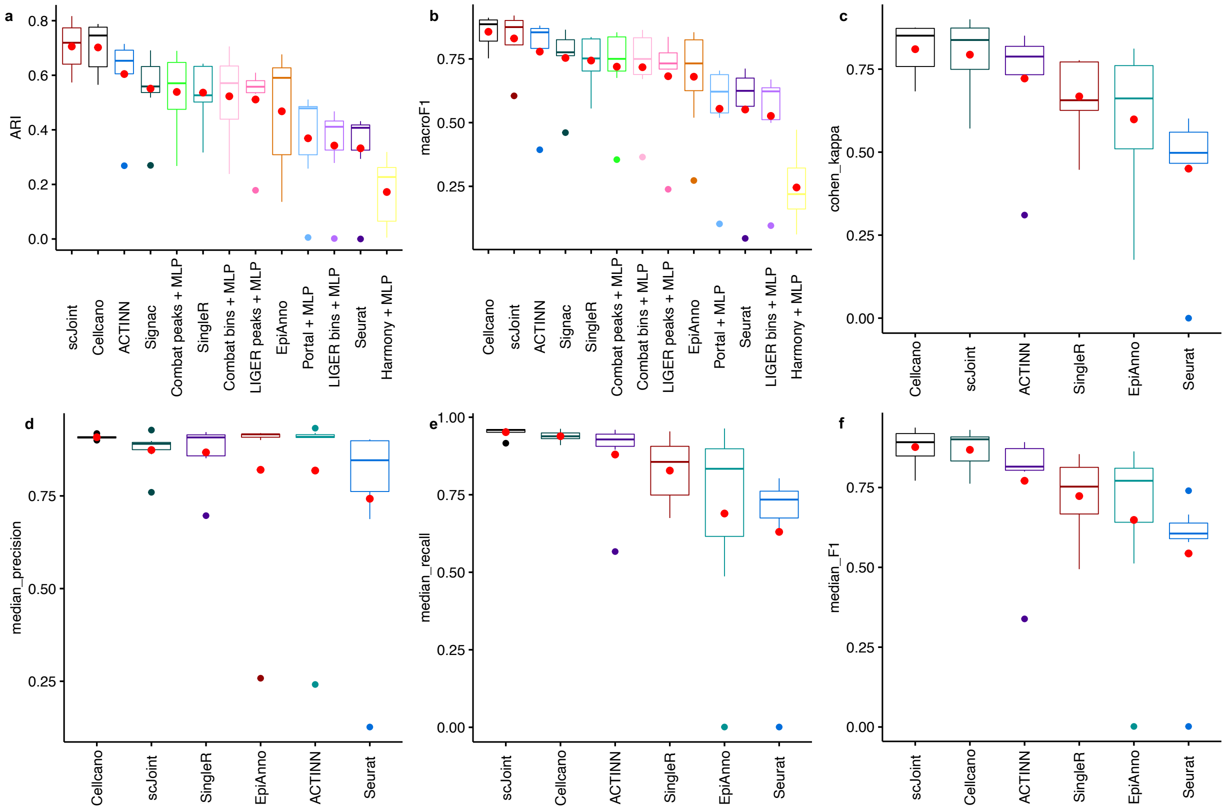

Supplementary Figure 3 Inside the boxes, the middle line indicates the median of the data while the bottom and upper lines indicate the 25th percentile and the 75th percentile of the data. Outside the boxes, the whiskers extend to the minimum and maximum values no greater than 1.5 times interquartile range. Those values outside the range are outliers, which are represented as dots with corresponding colors. Note that we use red dots to indicate the mean of the data. (**a**) ARI, (**b**) macroF1, (**c**) Cohen's kappa, (**d**) median precision, (**e**) median recall and (**f**) median F1 comparisons on $n = 7$ celltyping tasks using one human PBMCs FACS-sorted dataset as target. Each box contains $n = 7$ prediction results. (**a**)-(**b**) include prediction performances both from celltyping methods and integration with label transfer methods. All boxplots are ordered to have the leftmost method with highest average performance.

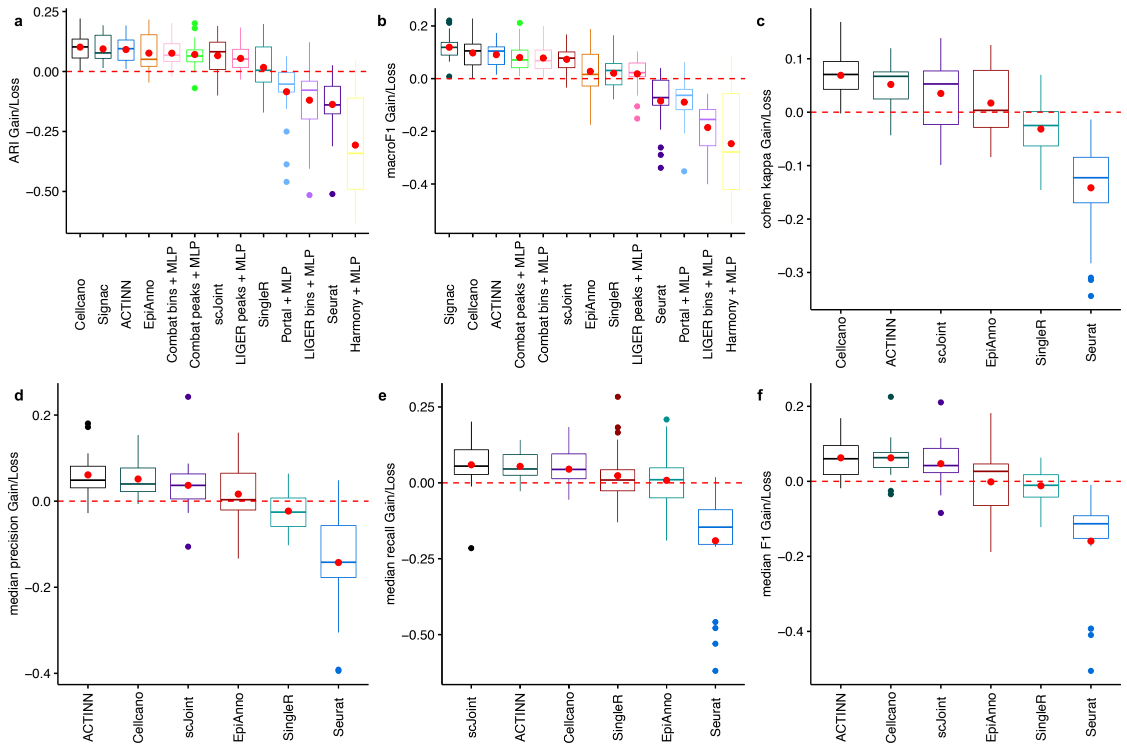

Supplementary Figure 4 Inside the boxes, the middle line indicates the median of the data while the bottom and upper lines indicate the 25th percentile and the 75th percentile of the data. Outside the boxes, the whiskers extend to the minimum and maximum values no greater than 1.5 times interquartile range. Those values outside the range are outliers, which are represented as dots with corresponding colors. Note that we use red dots to indicate the mean of the data. (**a**) ARI, (**b**) macroF1, (**c**) Cohen's kappa, (**d**) median precision, (**e**) median recall and (**f**) median F1 comparisons on $n = 22$ more human PBMCs celltyping tasks. Each box contains $n = 22$ prediction results. (**a**)-(**b**) include prediction performances both from celltyping methods and integration with label transfer methods. All boxplots are ordered to have the leftmost method with highest average performance.

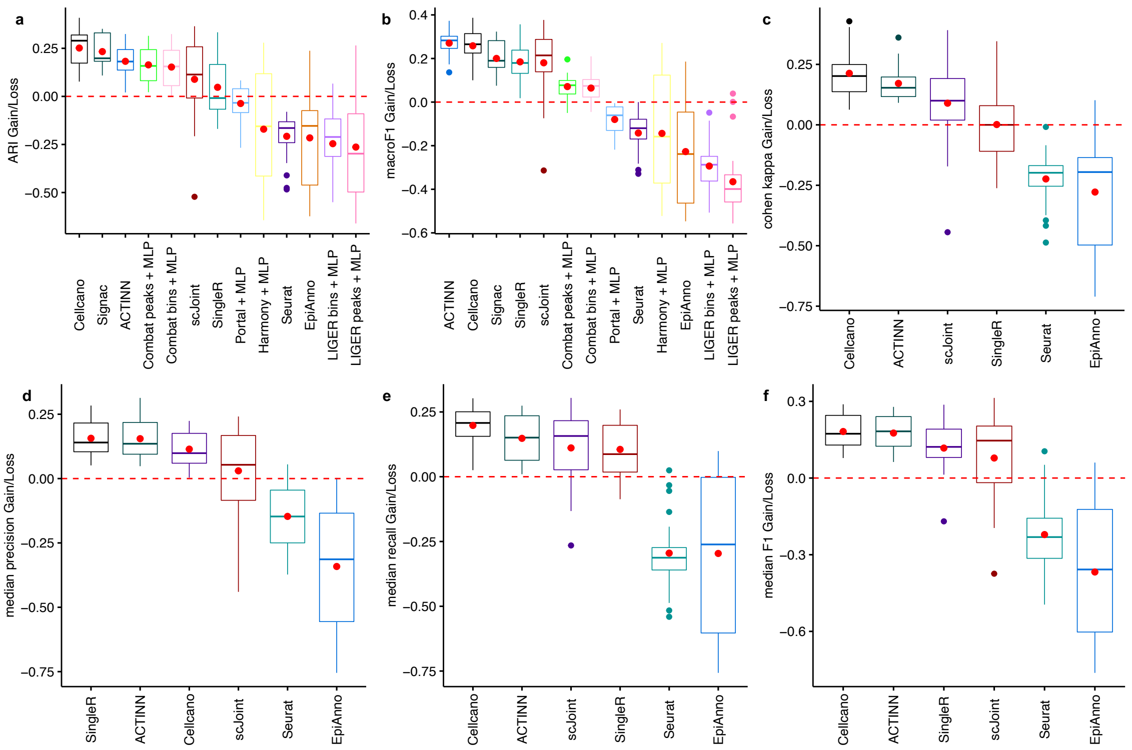

Supplementary Figure 5 Inside the boxes, the middle line indicates the median of the data while the bottom and upper lines indicate the 25th percentile and the 75th percentile of the data. Outside the boxes, the whiskers extend to the minimum and maximum values no greater than 1.5 times interquartile range. Those values outside the range are outliers, which are represented as dots with corresponding colors. Note that we use red dots to indicate the mean of the data. (**a**) ARI, (**b**) macroF1, (**c**) Cohen's kappa, (**d**) median precision, (**e**) median recall and (**f**) median F1 comparisons on $n = 21$ mouse brain celltyping tasks. Each box contains $n = 21$ prediction results. (**a**)-(**b**) include prediction performances both from celltyping methods and integration with label transfer methods. All boxplots are ordered to have the leftmost method with highest average performance.

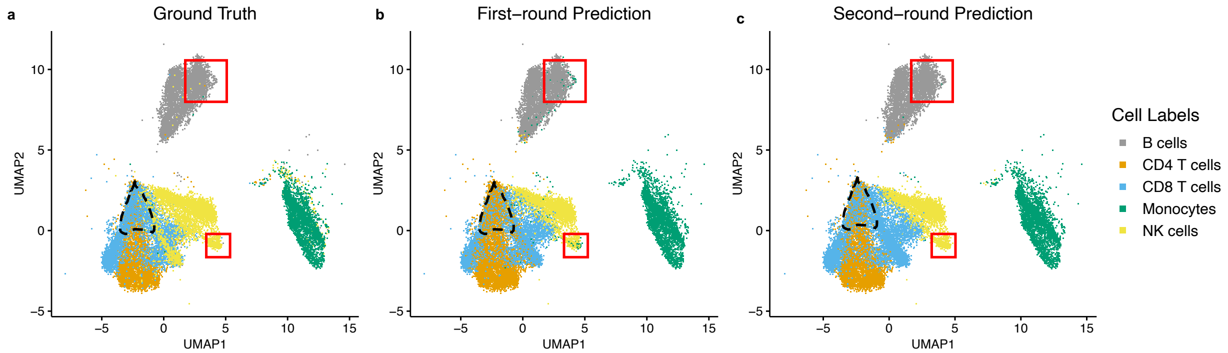

Supplementary Figure 6 UMAP visualization from one of the celltyping tasks using FACS-sorted dataset as target that contains $n = 21{,}214$ cells. The cells are colored with (**a**) ground truth labels; (**b)** Cellcano first-round predicted labels; and (**c**) Cellcano second-round predicted labels. The red boxes and black dotted line highlighted Cellcano's ability to correct wrongly assigned cells predicted from the first round.

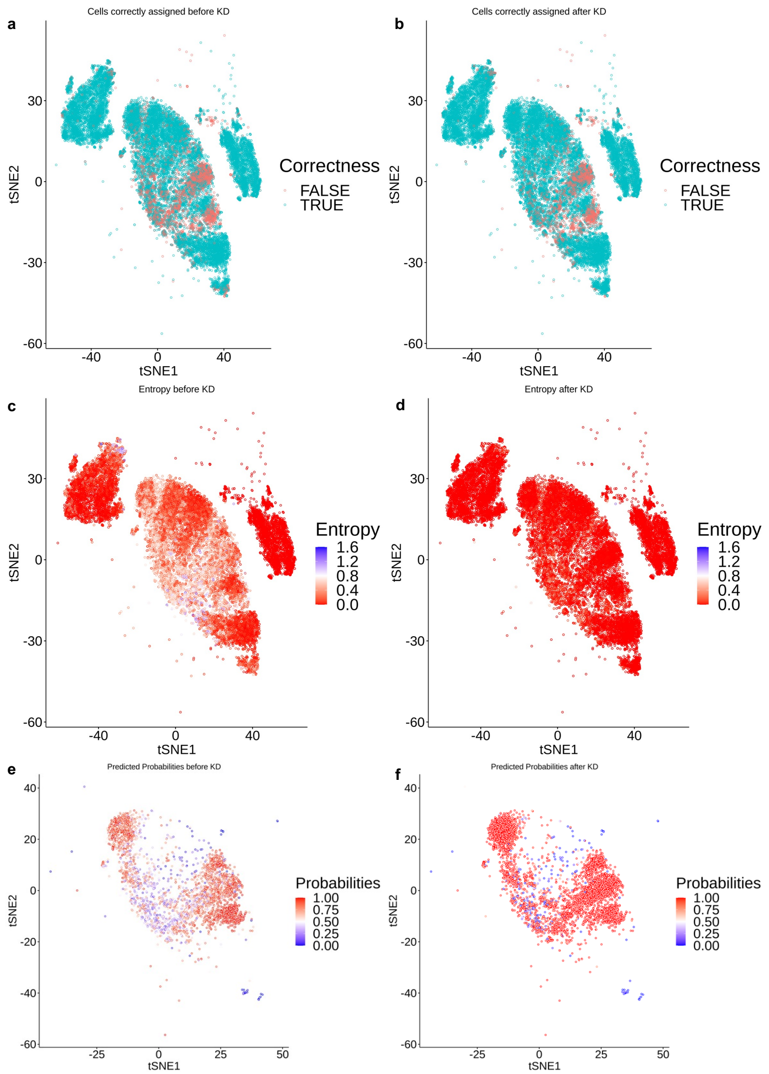

Supplementary Figure 7 Correctness, entropy, and predicted probabilities of CD8 T cells before and after second-round prediction. (**a**)-(**b**) visualize whether the cells are correctly predicted (**a**) before and (**b**) after second-round Knowledge Distillation (KD) model. (**c**)-(**d**) visualize whether entropies of cells (**c**) before and (**d**) after second-round KD model. Lower entropy shows more confidence in predicting the cell types. (**e**)-(**f**) separate CD8 T cells and show predicted probabilities (**e**) before and (**f**) after second-round KD model. Higher the predicted probabilities, more confident they are assigned as CD8 T cells.

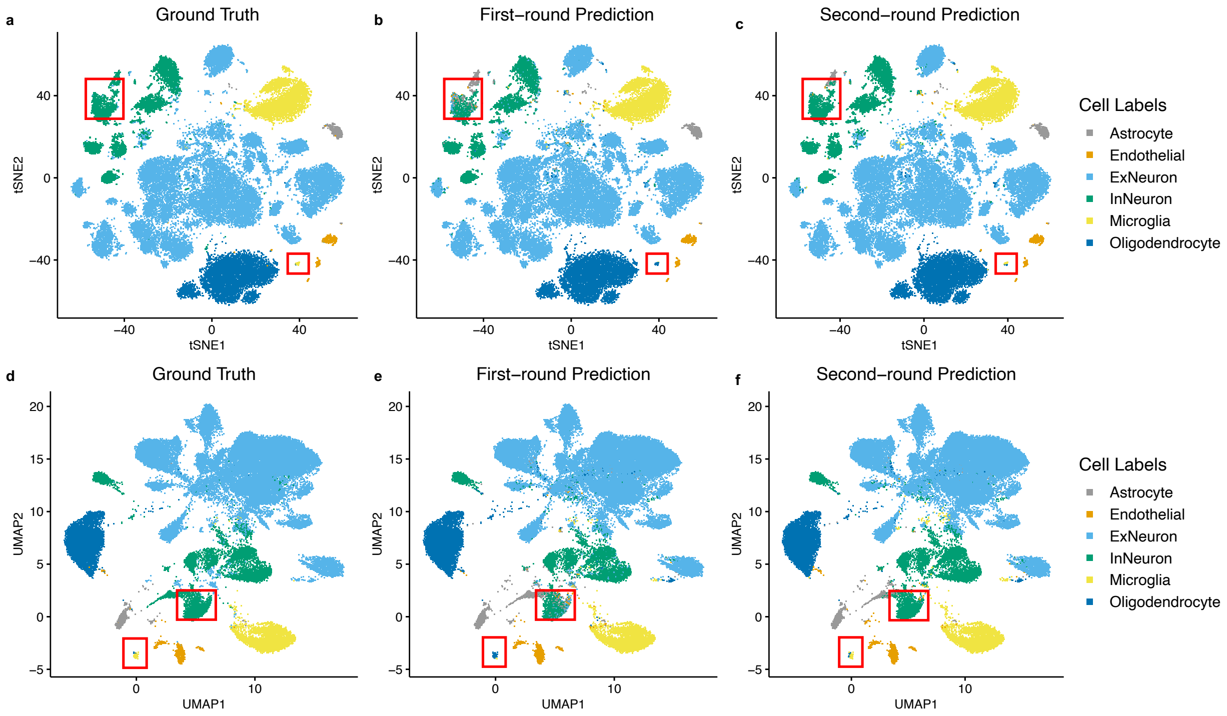

Supplementary Figure 8 Visualization showing prediction results on one of the celltyping tasks using all cells from Lareau et al. mouse brain datasets as target. (**a**)-(**c**) are tSNE visualizations and (**d**)-(**f**) are UMAP visualizations. The cells are colored with (**a**)(**d**) ground truth labels; (**b**)(**e**) Cellcano first-round predicted labels; and (**c**)(**f**) Cellcano second-round predicted labels. The red boxes indicate Cellcano's ability to correct wrongly assigned cells predicted from the first round.

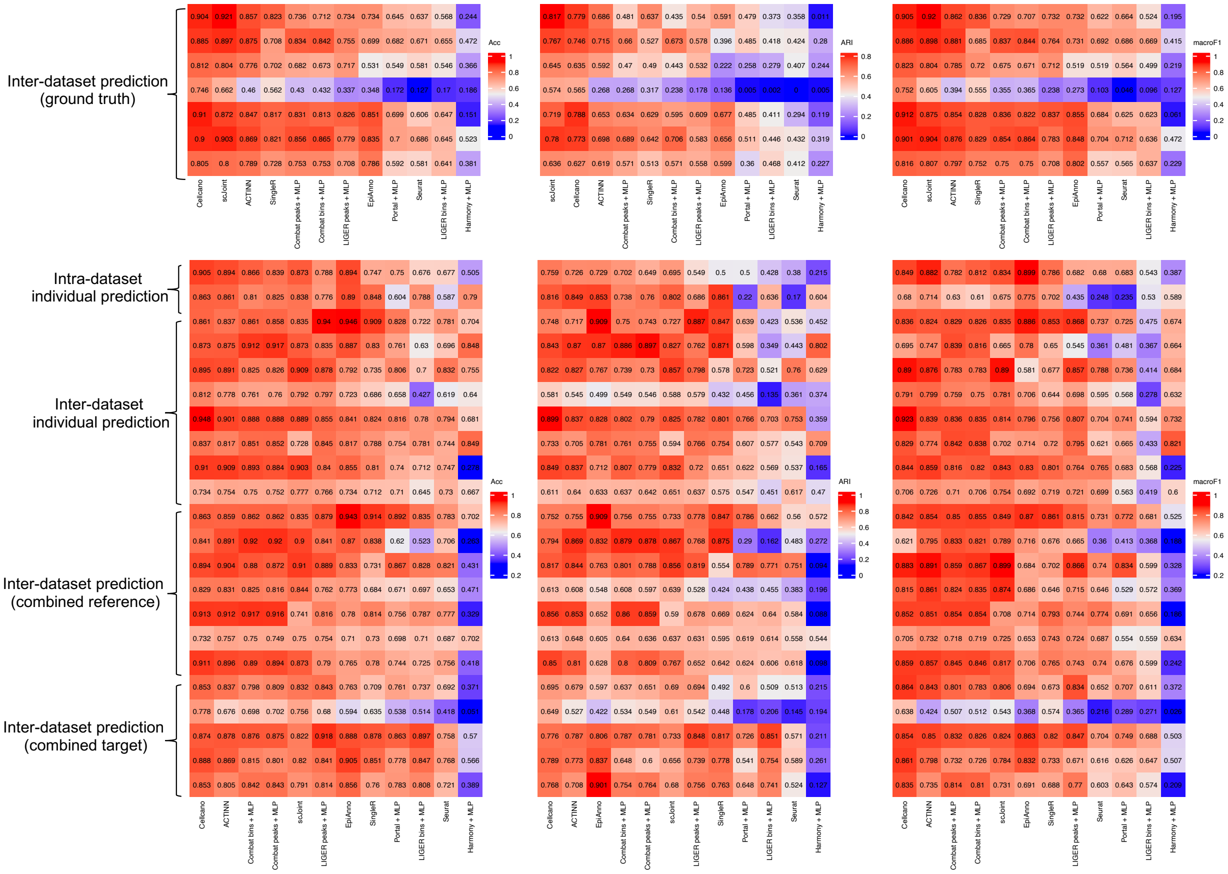

Supplementary Figure 9 Heatmap showing all prediction performances in 29 human PBMCs celltyping tasks. The celltyping tasks are labeled with corresponding categories. The heatmap is sorted to have the left most column with the highest average performance.

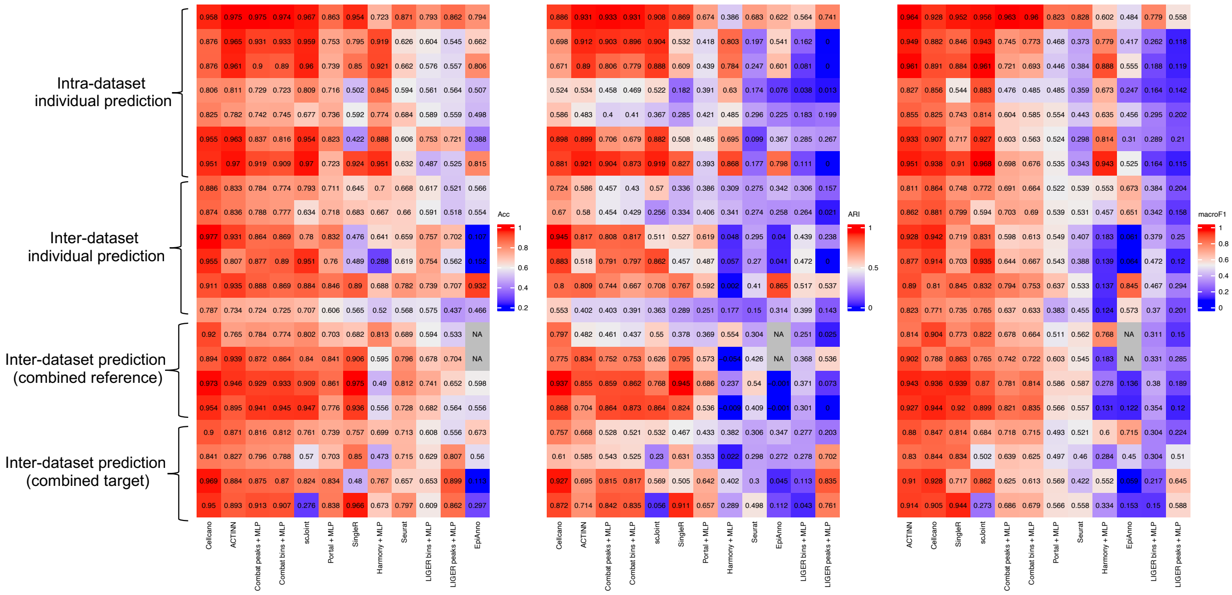

Supplementary Figure 10 Heatmap showing all prediction performances in 21 mouse brain celltyping tasks. The celltyping tasks are labeled with corresponding categories. The heatmap is sorted to have the left most column with the highest average performance. Note that EpiAnno fails to generate results for two larger celltyping tasks (denoted as NA in the figure) due to memory limit.

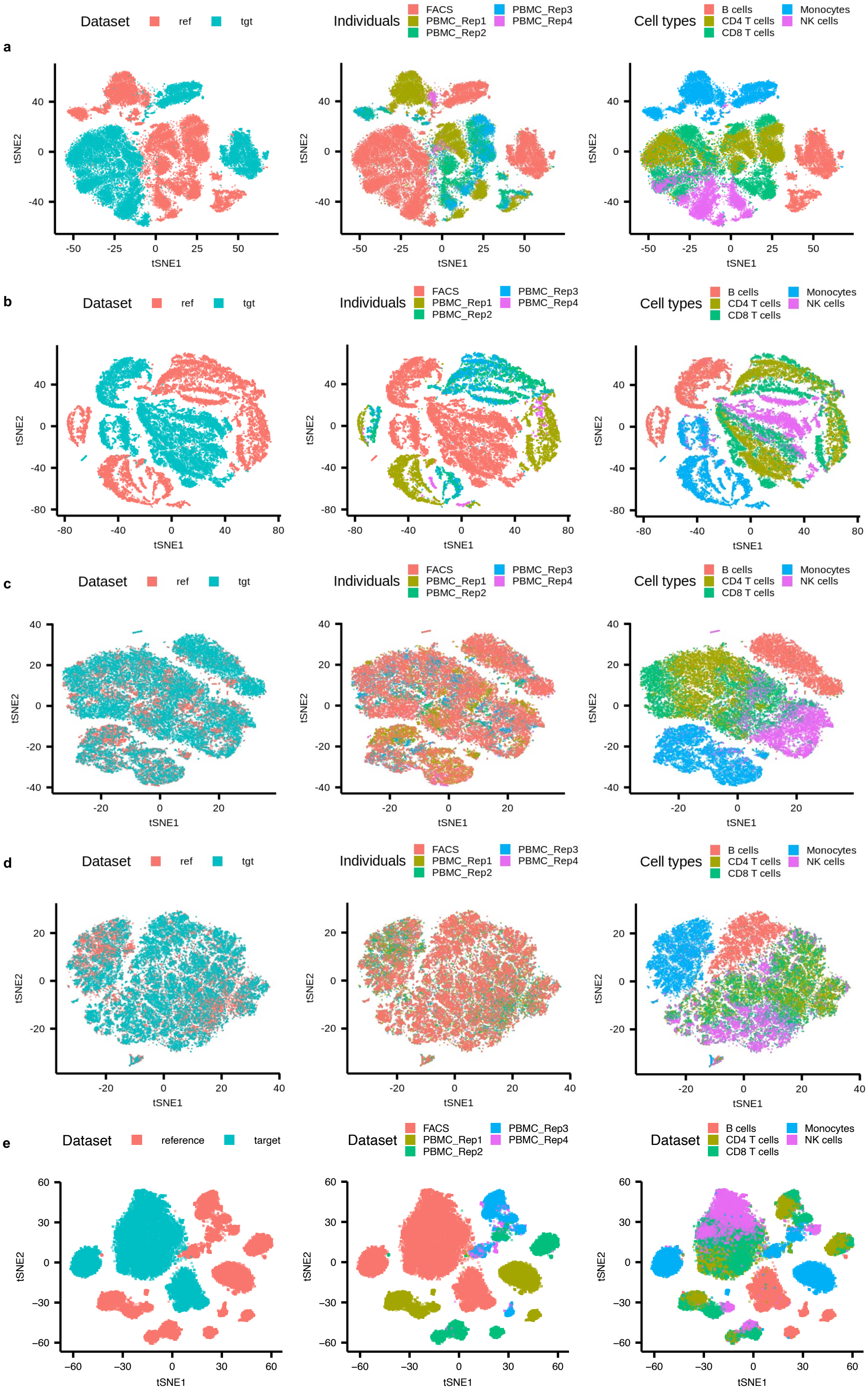
a
b
c
d
e
Dataset
ref
tgt
reference
target
Individuals
FACS
PBMC_Rep1
PBMC_Rep2
PBMC_Rep3
PBMC_Rep4
Cell types
B cells
CD4 T cells
CD8 T cells
Monocytes
NK cells
tSNE1
tSNE2

Supplementary Figure 11 Visualization on batch effect removal showing one of the celltyping tasks using one FACS-sorted dataset as target and a combination of four individuals from Satpathy et al. PBMCs dataset as reference. The left panels show the integrated datasets labeled by data source which is either from reference or target. The middle panels show the individual information, and the right panels show the cells colored by cell types. (**a**) shows the visualization before batch effect removal along with visualizations after batch effect removal conducted with (**b**) ComBat using peaks as input, (**c**) LIGER using peaks as input, (**d**) Portal using gene scores as input, and (**e**) Harmony using gene scores as input.

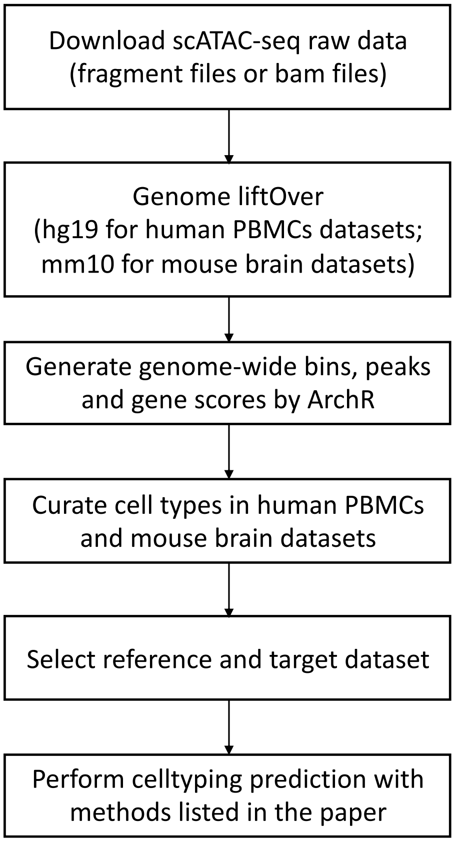

Supplementary Figure 12 A diagram shows our procedure of data preprocessing and data analysis.

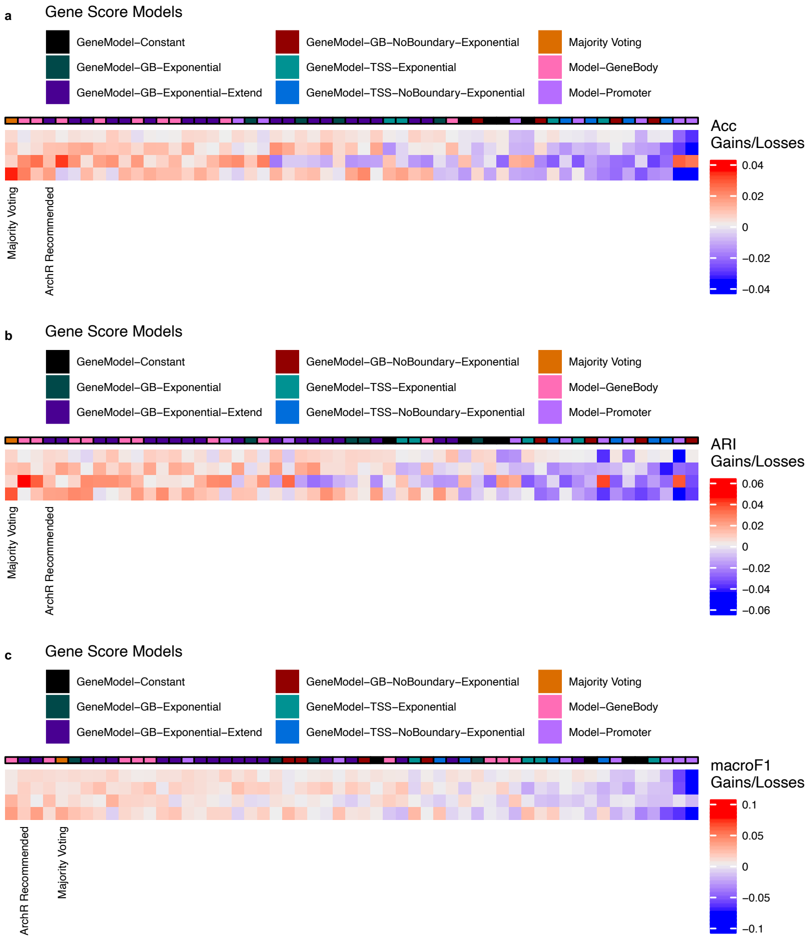

Supplementary Figure 13 (**a**) Acc, (**b**) ARI, and (**c**) macroF1 gains/losses on using different gene score models from four human PBMCs celltyping tasks. The columns are sorted by the average performance gains/losses. The gene model recommended by ArchR along with the majority voting result are labeled.

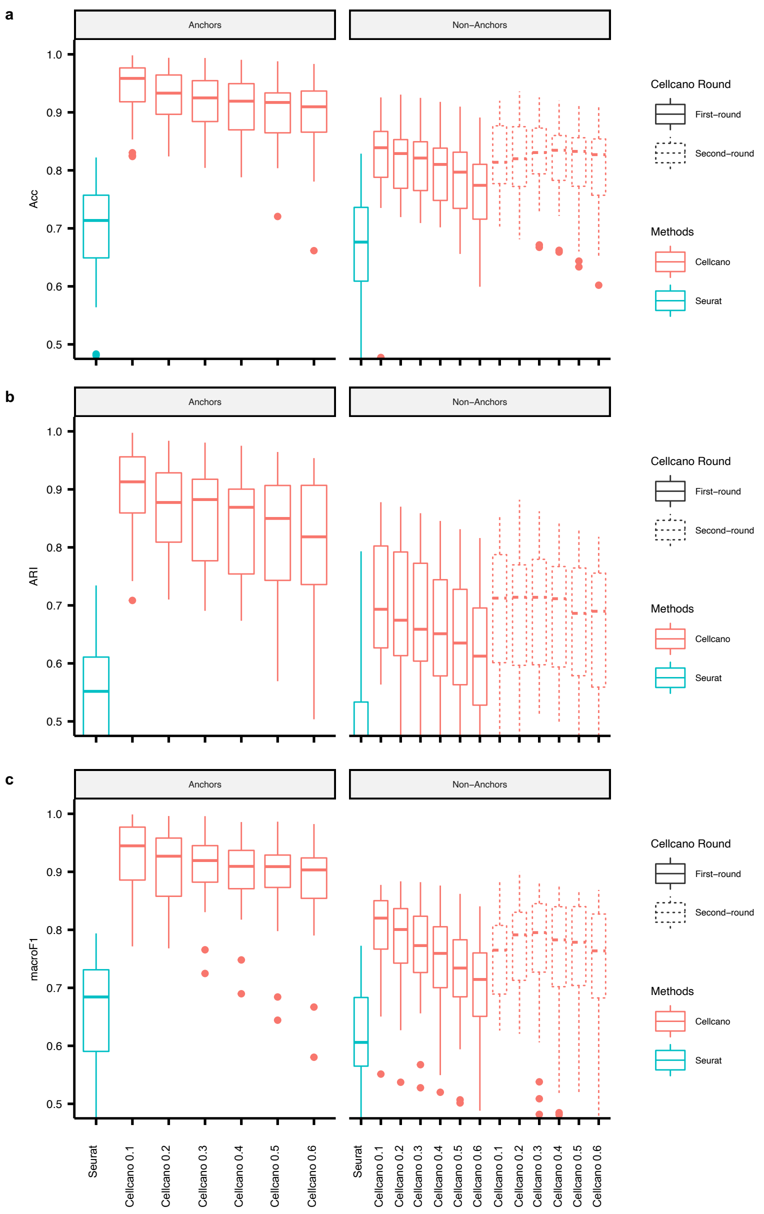

Supplementary Figure 14 Inside the boxes, the middle line indicates the median of the data while the bottom and upper lines indicate the 25th percentile and the 75th percentile of the data. Outside the boxes, the whiskers extend to the minimum and maximum values no greater than 1.5 times interquartile range. Those values outside the range are outliers, which are represented as dots with corresponding colors. (**a**) Accuracy, (**b**) ARI and (**c**) macroF1 values from anchors and non-anchors between Seurat and Cellcano using different entropy cutoffs in $n = 29$ human PBMCs celltyping tasks. Each box contains $n = 29$ prediction results. The left panels show the anchors performance, and the right panels show the non-anchors performance. In the right panels, the dotted boxplots show non-anchors performance after Cellcano's second-round prediction. The entropy quantile cutoffs are labeled on the x-axis.

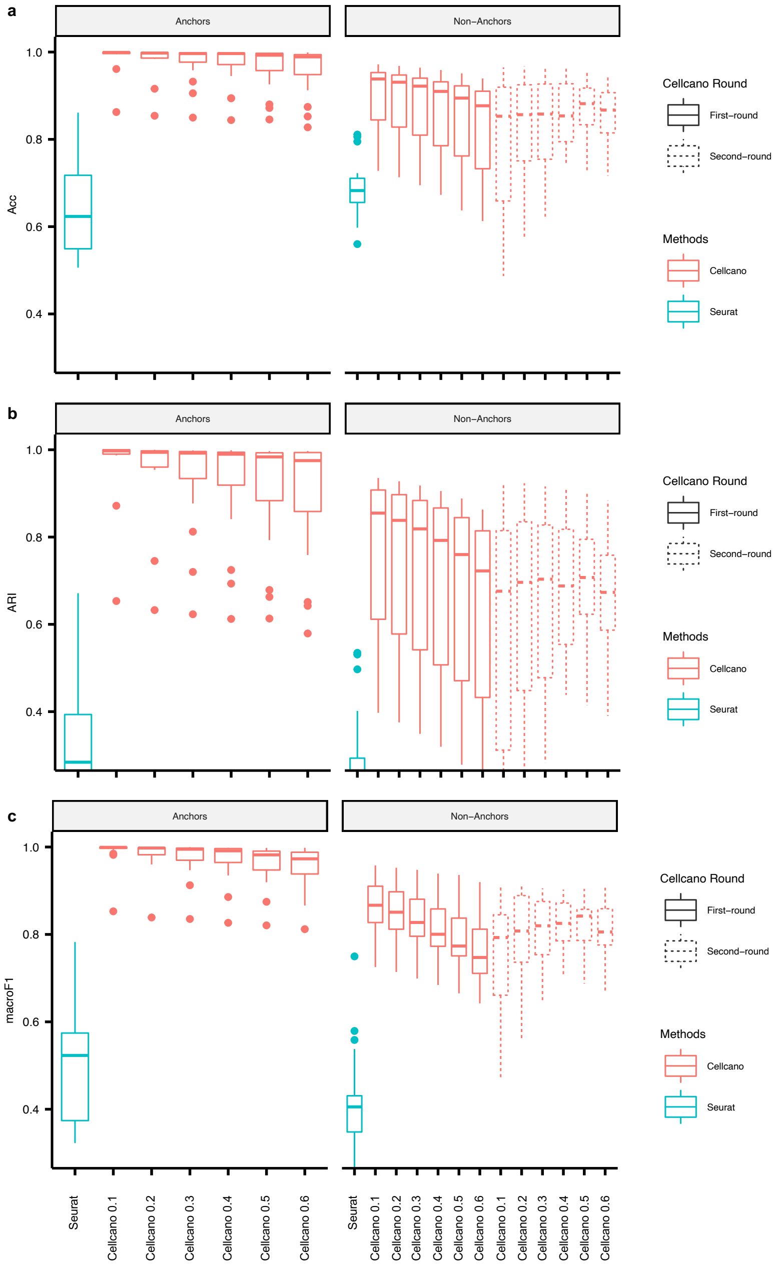

Supplementary Figure 15 Inside the boxes, the middle line indicates the median of the data while the bottom and upper lines indicate the 25th percentile and the 75th percentile of the data. Outside the boxes, the whiskers extend to the minimum and maximum values no greater than 1.5 times interquartile range. Those values outside the range are outliers, which are represented as dots with corresponding colors. (**a**) Accuracy, (**b**) ARI and (**c**) macroF1 values from anchors and non-anchors between Seurat and Cellcano using different entropy cutoffs in $n = 21$ mouse brain celltyping tasks. Each box contains $n = 21$ prediction results. The left panels show the anchors performance, and the right panels show the non-anchors performance. In the right panels, the dotted boxplots show non-anchors performance after Cellcano's second-round prediction. The entropy quantile cutoffs are labeled on the x-axis.

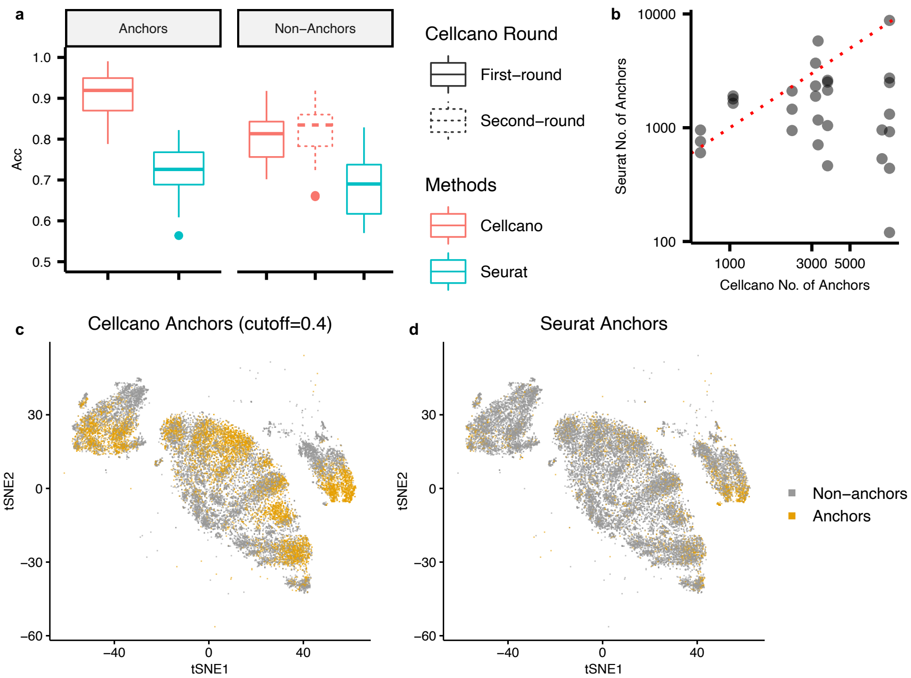

Supplementary Figure 16 Comparisons between Cellcano' anchors and Seurat's anchors. Inside the boxes, the middle line indicates the median of the data while the bottom and upper lines indicate the 25th percentile and the 75th percentile of the data. Outside the boxes, the whiskers extend to the minimum and maximum values no greater than 1.5 times interquartile range. Those values outside the range are outliers, which are represented as dots with corresponding colors. (**a**) Prediction accuracy comparison on anchors and non-anchors from Cellcano (red boxes) and Seurat (blue boxes) on $n = 29$ human PBMCs celltyping tasks. The dotted boxes show the performance of Non-Anchors from Cellcano second round's prediction. Here, we use entropy quantile cutoff as 0.4 to select anchors for Cellcano. Each box contains $n = 29$ prediction results. (**b**) Anchor number comparison from $n = 29$ human PBMCs celltyping tasks. (**c**)- (**d**) t-SNE plots showing one of the celltyping tasks using FACS-sorted dataset as target where (**c**) highlight anchors selected by Cellcano and (**d**) show anchors selected by Seurat.

Supplementary Notes

Supplementary Note 1: Details on designing celltyping tasks

In total, we designed 50 celltyping tasks involving different individuals as reference and target datasets from six datasets (four human PBMCs datasets and two mouse brain datasets). We design the celltyping tasks to mimic the following prediction scenarios:

- Intra-dataset individual prediction: users have one confidently annotated scATAC-seq profile from one individual and want to use it to annotate all other individuals from the same study.
- Inter-dataset individual prediction: users have one confidently annotated scATAC-seq profile from one individual and want to use it to annotate other individuals from different studies. In the mouse brain celltyping tasks, a special case is that we have tasks not only

for a different subject but also for a different brain region because mouse brain has several brain regions. We count them into this category.

- Inter-dataset prediction (combined reference): users have several well annotated scATAC-seq datasets and wish to use a large collection of public datasets to increase the reference data size and improve the prediction result. This is based on our previous research [5] where we found that combining individuals or datasets as reference could lead to better prediction results.
- Inter-dataset prediction (combined target): users have scATAC-seq data from multiple batches and want to determine their cell types in one run using a given reference.

We have one more task design which is Inter-dataset prediction (Ground truth) where we use the FACS-sorted human PBMCs dataset as target dataset. Since the FACS-sorted human PBMCs dataset can be considered as the ground truth, we use this category to better evaluate how Cellcano predicts compared to all other methods. However, this category will not appear in real cell type prediction scenario.

Supplementary Note 2: An introduction to different ArchR gene score models

The script to generate gene score models are provided by ArchR [6] (https://github.com/GreenleafLab/ArchR_2020). In total, there are eight categories of gene score models including:
(1) Model – Promoter: This class of models count the reads located on the promoter region with different window sizes.
(2) Model – GeneBody: This class of models count the reads located on the whole gene body with certain extension in up- or down-stream.
(3) GeneModel – Constant: This class of models count reads from 1K bps upstream transcription start site (TSS) and different bps downstream TSS. The constant gene model considers each read having the same weight as 1.
(4) GeneModel – TSS – Exponential: This class of models extract reads from 1K bps upstream and 100K bps downstream TSS. Gene boundaries are set so that reads from one gene body will not overlap with other gene bodies. Then, an exponential decay function is used to weight the reads from each windowed tile based on the distance to TSS. The exponential decay function is demonstrated as $\exp\left(-\frac{abs(distance)}{window} + \exp(-1)\right)$ with different *window* parameters.
(5) GeneModel – TSS – NoBoundary – Exponential: Same as (4) except no gene boundaries are set.
(6) GeneModel – GB – Exponential: Same as (4) except the distance in the exponential decay function is calculated based on the distance to gene bodies instead of TSS. Gene boundaries are set in this class of models.
(7) GeneModel – GB – Exponential – Extend: Same as (6) except the gene bodies are extended. The distance in the exponential decay function is calculated based on the extended gene bodies.
(8) GeneModel – GB – NoBoundary – Exponential: Same as (6) except there are no gene boundaries limitations.

The gene score model recommended by ArchR lies in category (7). It integrates the signals from the gene body with TSS extended 5kb in the upstream direction. Then, it weights the reads outside the gene body region and use the *window* parameter as 10,000.

Supplementary Note 3: Evaluation of using ArchR gene score model as input for Cellcano

When evaluating the choice of gene score model, we design four human PBMCs prediction celltyping tasks which are: (1) use PBMC_D10T1 from Granja et al. dataset as reference to predict PBMC_Rep1 from Satpathy et al. dataset; (2) use PBMC_D10T1 from Granja et al. dataset as reference to predict PBMC_Rep2 from Satpathy et al. dataset; (3) use PBMC_Rep1 from Stapathy et al. dataset as reference to predict PBMC_D10T1 from Granja et al. dataset; and (4) use PBMC_Rep1 from Satpathy et al. dataset as reference to predict PBMC_D11T1 from Granja et al. dataset.

The ArchR recommended gene score model resides in the "GeneModel – GB – Exponential – Extend" category, which covers signals on the whole gene body and adds bi-directional exponential decay weights on the reads outside the gene body area according to the distance to the gene body. It was shown in the original paper as the most accurate model to infer gene expression in matched scATAC-seq and scRNA-seq data. We then investigate whether using another model or applying a majority voting strategy with all ArchR 54 gene score models as input can result in a better prediction. To that end, for every celltyping task, we use each gene score as input in Cellcano to predict cell types, which results in 54 prediction results. Then, for the majority voting strategy, we take the one with the highest vote from all 54 predictions as the final predicted cell type. In total, we have 55 prediction results for each celltyping task.

Supplementary Figure 13a-c show the results from using all individual gene scores and the majority voting from four human PBMCs celltyping tasks. We again remove the baseline performance for each celltyping task to compute the gains/losses, and then order the heatmap to make the left column have the largest average gain. Overall, the top 10 or so performing gene score models are very similar. The majority voting Acc ranks the first, and the Acc of using recommended gene score model ranks the 4th (Supplementary Figure 13a). However, the average performance differences between majority voting and the ArchR recommended gene score model is very small (0.34%). Similar trends have been observed in ARI (majority voting ranks 1st and ArchR recommended gene score model ranks 4th, Supplementary Figure 13b) and macroF1 (ArchR recommended gene score model ranks 2nd and majority voting ranks 5th, Supplementary Figure 13c). In summary, the slight improvement of Acc and ARI in majority voting, which uses 54 times computational resources, is unworthy. Moreover, since the ArchR recommended gene score has very similar results and shows good performances in other tasks, we recommend using it as Cellcano's input.

Supplementary Note 4: Exploration on numbers of Cellcano's anchors selected

We use different entropy quantile cutoffs (0.1 to 0.6 with step size 0.1) to select different number of anchors in human PBMCs celltyping tasks and mouse brain celltyping tasks. Based on the ground-truth labels, we evaluate the performances between anchors and non-anchors (Supplementary Figure 14a-c, Supplementary Figure 15a-c). Since we are inspired by Seurat, we also add it into comparisons. However, when comparing between Seurat and Cellcano, Seurat's anchors and non-anchors do not outperform any anchors or non-anchors selected by Cellcano based on different entropy quantile cutoffs. We then focus on analyzing Cellcano's results and

observe that when the quantile cutoff is lower, the anchor accuracies are higher. This is as expected because a more stringent confidence criterion will lead to higher prediction accuracy. However, using fewer anchors means the training dataset is smaller in the second round. Moreover, using too few anchors could fail to capture the full scope of target data distribution since the most confident cells tend to cluster around cluster centroids. Both can result in decreased performance of non-anchors when performing second-round prediction (Supplementary Figure 14-15, right panels where the entropy quantile cutoffs are 0.1 and 0.6). On the other hand, choosing too many anchors will include many wrongly predicted cells, which is detrimental to the second-round model training. Our exploration shows that the final prediction performance depends on a balance between anchor numbers and anchor accuracy.

Supplementary Note 5: Comparison between anchors selected by Cellcano with 0.4 as entropy quantile cutoff and anchors selected by Seurat

Similar to Seurat, Cellcano selects anchors from the target dataset and uses them as reference to predict cell types for non-anchors in the second round. However, the procedure for anchor selection in Cellcano is very different. Seurat uses Mutual Nearest Neighbors (MNN) in a low-dimensional space determined by canonical component analysis (CCA) to select anchors, which relies on the linear relationship between reference and target. The number of anchors selected is further determined by the parameter of how many neighbors are examined. Differently, Cellcano obtains predicted probabilities for cells in target data from the first-round MLP, and then selects anchors based on the prediction entropies.

According to our exploration, 0.4 is an appropriate entropy quantile cutoff for selecting anchors. We therefore compare the performances between anchors selected by Cellcano and anchors selected by Seurat. For all 29 human PBMCs celltyping tasks, Cellcano's anchors achieve much higher accuracy (median: 91.93% and mean: 91.04%) compared to Seurat (median: 71.36% and mean: 69.04%), even though Cellcano selects more anchors (Supplementary Figure 16a-b). We also compare the non-anchors performances in Cellcano before and after the second-round prediction and observe an increase of 2.44% in median and 3.27% in mean. The improvement further validates the usefulness of Cellcano's two-round prediction procedure. We then use one celltyping task (one FACS-sorted human PBMCs dataset as target, a combination of four individuals as reference) as an example to visualize the anchors selected by Cellcano (Supplementary Figure 16c-d). We conclude that anchors selected by Cellcano can better capture the full scope of target data distribution (Supplementary Figure 16c) compared to those selected by Seurat (Supplementary Figure 16d).

Supplementary Note 6: Details on datasets processing

We download either fragment or bam files for all datasets. We collect datasets for human PBMCs, and mouse brains listed in Supplementary Table 1.

Datasets in human PBMCs include:

- The Satpathy et al. PBMC dataset [1] is downloaded from GEO with the accession number GSE129785. It contains 4 healthy individuals labeled as PBMC_Rep1, PBMC_Rep2,

PBMC_Rep3, and PBMC_Rep4. We download the fragment files for them. The cell types are annotated based on unsupervised clustering with prior biological knowledge.

- The Granja et al. PBMC dataset [2] is from a mixed-phenotype acute leukemias study (MPAL). We download the fragment files from GEO with the accession number GSE139369. We focus on the 5 replicates which contain 3 healthy donors: PBMC_D10T1, PBMC_D11T1, PBMC_D12T1, PBMC_D12T2, and PBMC_D12T3. The cell types are annotated based on Seurat SNN clustering results as well as the manually curated marker gene lists.
- The 10X PBMC dataset is downloaded from the 10X Single Cell Multiome ATAC + Gene Expression with granulocytes removed through cell sorting. We use the data with 10k cells. The dataset contains one healthy donor and the cell type annotations are obtained from MOFA pipeline [7].
- The FACS PBMC dataset [3] is available on GEO with accession number as GSE123578. Five human PBMCs cell types are sorted: CD4 T cells, CD8 T cells, B cells, Monocytes, and NK cells.

All human PBMCs datasets are mapped to human genome build hg19, except for 10X PBMC dataset, which is based on hg38. We use liftOver to map that dataset to hg19 so that all four datasets are consistent. All cell types are curated into 6 major cell types: B cells, CD4 T cells, CD8 T cells, NK cells, Monocytes and Dendritic cells.

The mouse brain datasets include:

- The Lareau et al. dataset [3] is downloaded from GEO with accession number as GSE123581. There are 2 mice in this dataset. Cell types are labeled based on the projection of another scRNA-seq mouse brain dataset. The projection is done by calculating the correlation between the promoter-region chromatin accessibility scores and gene expression on marker genes.
- The Cusanovich et al. dataset [4] is obtained from The Mouse sci-ATAC-seq Atlas (https://atlas.gs.washington.edu/mouse-atac/data/). We extract WholeBrainA_62216, WholeBrainA_62816, PreFrontalCortex_62216 and Cerebellum_62216 as our mouse brain samples. Cells are annotated based on unsupervised clustering and cluster-specific marker gene lists.

All mouse brain datasets are mapped to mouse genome build mm10, except for the dscATAC-seq Mouse Brain dataset, which is based on mm9. We use liftOver to lift the genome to mm10. We curate all cells into 7 major cell types including: Excitatory neurons, Inhibitory neurons, Microglia, Endothelial, Astrocyte, Oligodendrocyte and Polydendrocyte.

References

1. Satpathy, A. T. *et al.* Massively parallel single-cell chromatin landscapes of human immune cell development and intratumoral T cell exhaustion. *Nat Biotechnol* **37**, 925–936 (2019).

2. Granja, J. M. *et al.* Single-cell multiomic analysis identifies regulatory programs in mixed-phenotype acute leukemia. *Nat Biotechnol* **37**, 1458–1465 (2019).

3. Lareau, C. A. *et al.* Droplet-based combinatorial indexing for massive-scale single-cell chromatin accessibility. *Nat Biotechnol* **37**, 916–924 (2019).

4. Cusanovich, D. A. *et al.* A Single-Cell Atlas of In Vivo Mammalian Chromatin Accessibility. *Cell* **174**, 1309-1324.e18 (2018).

5. Ma, W., Su, K. & Wu, H. Evaluation of some aspects in supervised cell type identification for single-cell RNA-seq: classifier, feature selection, and reference construction. *Genome Biology* **22**, 264 (2021).

6. Granja, J. M. *et al.* ArchR is a scalable software package for integrative single-cell chromatin accessibility analysis. *Nat Genet* **53**, 403–411 (2021).

7. Multi-Omics Factor Analysis—a framework for unsupervised integration of multi-omics data sets. *Molecular Systems Biology* **14**, e8124 (2018).
