# Supplementary material for: Cellcano: supervised cell type identification for single cell ATAC-seq data
Source: Nat Commun. 2023 Apr 3;14:1864. doi: 10.1038/s41467-023-37439-3 (PMC10070275; doi:10.1038/s41467-023-37439-3)
Supplement: Supplementary file 3 — Description of Additional Supplementary Files [file 41467_2023_37439_MOESM3_ESM.pdf]

# Description of Additional Supplementary Files

**Title:** Supplementary Dataset 1

**Description:** Supplementary table describes 29 human PBMCs celltyping tasks designed for this study.

**Title:** Supplementary Dataset 2

**Description:** Supplementary table describes 21 mouse brain celltyping tasks designed for this study.
