# Supplementary material for: Cellcano: supervised cell type identification for single cell ATAC-seq data
Source: Nat Commun. 2023 Apr 3;14:1864. doi: 10.1038/s41467-023-37439-3 (PMC10070275; doi:10.1038/s41467-023-37439-3)
Supplement: Supplementary file 6 — Reporting Summary [file 41467_2023_37439_MOESM6_ESM.pdf]

nature portfolio

Corresponding author(s): Dr. Hao Wu

Last updated by author(s): Mar 2, 2023

Reporting Summary

Nature Portfolio wishes to improve the reproducibility of the work that we publish. This form provides structure for consistency and transparency in reporting. For further information on Nature Portfolio policies, see our Editorial Policies and the Editorial Policy Checklist.

Statistics

For all statistical analyses, confirm that the following items are present in the figure legend, table legend, main text, or Methods section.

n/a	Confirmed	
☐	☒	The exact sample size (n) for each experimental group/condition, given as a discrete number and unit of measurement
☒	☐	A statement on whether measurements were taken from distinct samples or whether the same sample was measured repeatedly
☐	☒	The statistical test(s) used AND whether they are one- or two-sided<br>*Only common tests should be described solely by name; describe more complex techniques in the Methods section.*
☒	☐	A description of all covariates tested
☒	☐	A description of any assumptions or corrections, such as tests of normality and adjustment for multiple comparisons
☐	☒	A full description of the statistical parameters including central tendency (e.g. means) or other basic estimates (e.g. regression coefficient) AND variation (e.g. standard deviation) or associated estimates of uncertainty (e.g. confidence intervals)
☐	☒	For null hypothesis testing, the test statistic (e.g. F, t, r) with confidence intervals, effect sizes, degrees of freedom and P value noted<br>*Give P values as exact values whenever suitable.*
☒	☐	For Bayesian analysis, information on the choice of priors and Markov chain Monte Carlo settings
☒	☐	For hierarchical and complex designs, identification of the appropriate level for tests and full reporting of outcomes
☒	☐	Estimates of effect sizes (e.g. Cohen's d, Pearson's r), indicating how they were calculated

Our web collection on statistics for biologists contains articles on many of the points above.

Software and code

Policy information about availability of computer code

Data collection	All data analyzed within this manuscript are publicly available. No additional software was used to collect data.
Data analysis	Cellcano code is publicly available on Github (https://github.com/marvinquiet/Cellcano) and it is also publicly available on PyPI with the latest version as v1.0.4 (https://pypi.org/project/Cellcano/). Cellcano python package is built upon Python and the recommended version is v3.8. There are several Python package dependencies, including tensorflow (v2.7.1), anndata (v0.7.4), scanpy (v1.8.2), numpy (v1.19.2), h5py (v2.10.0), keras and rpy2. R environment and the R package ArchR (v1.0.1) are also suggested to be installed for generating gene scores.<br><br>For the raw scATAC-seq data, ArchR (v1.0.1, https://www.archrproject.com/index.html) is used to generate gene score matrices and peak count matrices. We have benchmarked our Cellcano method to other computational celltyping tools including: Seurat (v4.3.0, https://satijalab.org/seurat/), Signac (v1.7.0, https://stuartlab.org/signac/index.html), scJoint (https://github.com/SydneyBioX/scJoint), SingleR (v2.0.0, https://bioconductor.org/packages/release/bioc/html/SingleR.html), ACTINN (https://github.com/mafeiyang/ACTINN) and EpiAnno (v1.0.0, https://github.com/xy-chen16/EpiAnno). In the meantime, we also compare our Cellcano method to other integration methods with label transfer. These integration tools include LIGER (v1.0.0, https://github.com/welch-lab/liger), ComBat (provided by Scanpy v1.8.2, https://scanpy.readthedocs.io/en/stable/generated/scanpy.pp.combat.html), Harmony (v0.1.0, https://portals.broadinstitute.org/harmony/) and Portal (v1.0.2, https://github.com/YangLabHKUST/Portal).

For manuscripts utilizing custom algorithms or software that are central to the research but not yet described in published literature, software must be made available to editors and reviewers. We strongly encourage code deposition in a community repository (e.g. GitHub). See the Nature Portfolio guidelines for submitting code & software for further information.

Data

Policy information about availability of data

All manuscripts must include a data availability statement. This statement should provide the following information, where applicable:

- Accession codes, unique identifiers, or web links for publicly available datasets
- A description of any restrictions on data availability
- For clinical datasets or third party data, please ensure that the statement adheres to our policy

All datasets are publicly available, and the access numbers or the downloaded websites are provided by the original publications.
1. The Satpathy et al. data used in this study is available in the Gene Expression Omnibus (GEO) dataset under accession code GSE129785 [https://www.ncbi.nlm.nih.gov/geo/query/acc.cgi?acc=GSE129785].
2. The Granja et al. data is available in the GEO dataset under accession code GSE139369 [https://www.ncbi.nlm.nih.gov/geo/query/acc.cgi?acc=GSE139369].
3.The 10X PBMCs data is available in the 10X genomics datasets. We downloaded the raw data under the Single Cell Multiome ATAC + Gene Expression category named PBMC from a Healthy Donor – Granulocytes Removed Through Cell Sorting (10K) processed by Cell Ranger ARC 2.0.0 [https://www.10xgenomics.com/resources/datasets/pbmc-from-a-healthy-donor-granulocytes-removed-through-cell-sorting-10-k-1-standard-2-0-0].
4.The FACS PBMCs data is available in the GEO dataset under accession code GSE123578 [https://www.ncbi.nlm.nih.gov/geo/query/acc.cgi?acc=GSE123578].
5.The Lareau et al. data is available in the GEO dataset under accession code GSE123581 [https://www.ncbi.nlm.nih.gov/geo/query/acc.cgi?acc=GSE123581].
6.The Cusanovich et al. data is available in the Mouse Atlas dataset under Downloads tab [https://atlas.gs.washington.edu/mouse-atac/data/].

The liftOver chain files to convert human genome build hg38 to hg19 data is available in the UCSC file server saved as hg38ToHg19.over.chain.gz [https://hgdownload.cse.ucsc.edu/goldenpath/hg38/liftOver/] and the chain files to convert mouse genome build mm9 to mm10 data is available in the USCS file server saved as mm9ToMm10.over.chain.gz [https://hgdownload.cse.ucsc.edu/goldenpath/mm9/liftOver/].

Human research participants

Policy information about studies involving human research participants and Sex and Gender in Research.

Reporting on sex and gender	N/A. There's no sex or gender information in the data.
Population characteristics	N/A. There's no population characteristics information in the data.
Recruitment	N/A. All data are public. We did not perform recruitment.
Ethics oversight	N/A.

Note that full information on the approval of the study protocol must also be provided in the manuscript.

Field-specific reporting

Please select the one below that is the best fit for your research. If you are not sure, read the appropriate sections before making your selection.

☒ Life sciences ☐ Behavioural & social sciences ☐ Ecological, evolutionary & environmental sciences

For a reference copy of the document with all sections, see nature.com/documents/nr-reporting-summary-flat.pdf

Life sciences study design

All studies must disclose on these points even when the disclosure is negative.

Sample size	We collected 6 high-quality scATAC-seq datasets, where 4 datasets from human PBMCs system and 2 dataset from mouse brain systems. In human PBMCs datasets, there are 9 different individuals and in the mouse brain datasets, there are 4 different individuals. In human PBMCs dataset, one dataset is obtained is from FACS-sorted technique which can serve as ground truth.<br><br>We select reference and target datasets from these individuals as reference and target to perform our celltyping prediction tasks. The task design aims to mimic the real prediction scenarios and more details are provided in Supplementary Note 1.<br><br>No statistical method was used to predetermine sample size.
Data exclusions	We first collected the raw data and then used ArchR to process them into gene scores. In ArchR, there are two quality control parameters minTSS and minFrags as exclusion criteria to exclude low-quality cells. We set the two parameters according to the descriptions of original dataset papers, which follows the standard procedure.
Replication	We directly collected the publicly available datasets and the replicates were decided by the original dataset papers.<br>As for the results reproducibility, we set the same random seed for python random package and tensorflow package. Therefore, we can assure that our results are reproducible.

Randomization	The experiments were not randomized.
Blinding	The Investigators were not blinded to allocation during experiments and outcome assessment.

Reporting for specific materials, systems and methods

We require information from authors about some types of materials, experimental systems and methods used in many studies. Here, indicate whether each material, system or method listed is relevant to your study. If you are not sure if a list item applies to your research, read the appropriate section before selecting a response.

Materials & experimental systems

n/a	Involved in the study
☒	☐ Antibodies
☒	☐ Eukaryotic cell lines
☒	☐ Palaeontology and archaeology
☒	☐ Animals and other organisms
☒	☐ Clinical data
☒	☐ Dual use research of concern

Methods

n/a	Involved in the study
☒	☐ ChIP-seq
☒	☐ Flow cytometry
☒	☐ MRI-based neuroimaging
